# Disentangling urban habitat and matrix effects on wild bee species

**DOI:** 10.7717/peerj.2729

**Published:** 2016-11-29

**Authors:** Leonie K. Fischer, Julia Eichfeld, Ingo Kowarik, Sascha Buchholz

**Affiliations:** 1 Department of Ecology, Technische Universität Berlin, Berlin, Germany; 2 Berlin-Brandenburg Institute of Advanced Biodiversity Research (BBIB), Berlin, Germany

**Keywords:** Apidae, Ecological restoration, Habitat management, Hymenoptera, Pollinators, Urban green infrastructure, Urbanization

## Abstract

In face of a dramatic decline of wild bee species in many rural landscapes, potential conservation functions of urban areas gain importance. Yet effects of urbanization on pollinators, and in particular on wild bees, remain ambiguous and not comprehensively understood. This is especially true for amenity grassland and extensively managed wastelands within large-scale residential housing areas. Using Berlin as a study region, we aimed to investigate (a) if these greenspaces are accepted by wild bee assemblages as foraging habitats; (b) how assemblage structure of bees and individual bee species are affected by different habitat (e.g., management, flower density) and urban matrix variables (e.g., isolation, urbanization); and (c) to what extent grassland restoration can promote bees in urban environments. In summer 2012, we collected 62 bee species belonging to more than 20% of the taxa known for Berlin. Urbanization significantly affected species composition of bees; 18 species were affiliated to different levels of urbanization. Most bee species were not affected by any of the environmental variables tested, and urbanization had a negative effect only for one bee species. Further, we determined that restoration of diverse grasslands positively affected bee species richnesss in urban environments. We conclude that differently structured and managed greenspaces in large-scale housing areas can provide additional foraging habitats and refuges for pollinators. This supports approaches towards a biodiversity friendly management within urban regions and may be of particular importance given that anthropogenic pressure is increasing in many rural landscapes.

## Introduction

The observed dramatic decline of wild bee species richness and abundances during the last decades (Potts et al., 2010; Carvalheiro et al., 2013)—especially in rural landscapes—is of great concern due to the enormous agricultural, ecological and cultural value (Gallai et al., 2009; Ollerton, Winfree & Tarrant, 2011) of bees. Unlike other ‘less’ appealing pollinator species, bees can be considered as flagship species that raise the awareness of city-dwellers to biodiversity and therefore, play an important role within the field of nature conservation (Fortel et al., 2014).

Rural landscapes undergo wide-ranging transformations, such as intensive farming including monocultures and in general the loss of natural or semi-natural habitats causing adverse effects for many insects (e.g., Kennedy et al., 2013 for bees; Santos de Araújo, Tscharntke & Almeida-Neto, 2015 for herbivorous insects in general). At the same time, it is widely assumed that cities can provide suitable secondary habitats for a variety of invertebrate species (Lundholm & Richardson, 2010; Jones & Leather, 2012; Shwartz et al., 2014). This is because urban areas offer a unique ecological environment with warm climate and high habitat diversity leading to diverse nesting and foraging opportunities (Ahrné, Bengtsson & Elmqvist, 2009; Sattler et al., 2010). Furthermore, plant species richness is usually higher in urban than in rural areas (Kühn, Brandl & Klotz, 2004) and flowers—which are less often treated with pesticides than flowers in agricultural areas (Hostetler & McIntyre, 2001)—are available abundantly throughout the year (Fetridge, Ascher & Langellotto, 2008).

However, a deeper understanding of how cities can help mitigate biodiversity loss in rural landscapes is urgently needed, especially for pollinators (Banaszak-Cibicka & Żmihorski, 2012; Baldock et al., 2015). Effects of urbanization on insects in general and pollinators in particular remain ambiguous and are not comprehensively understood (Threlfall et al., 2015). Existing studies report positive as well as negative effects whereas the latter seem to prevail (McKinney, 2006; McKinney, 2008). While the diversity of pollinators in urban environments has been addressed in a range of recent studies (e.g., Banaszak-Cibicka & Żmihorski, 2012; Baldock et al., 2015; Hülsmann et al., 2015; Threlfall et al., 2015), many of these focussed on a classical range of urban green spaces (e.g., cemeteries, gardens, parks).

This is a drawback because conservation in urban areas demands the inclusion of the whole range of urban ecosystems (Hobbs, Higgs & Harris, 2009; Kowarik, 2011) including informal greenspaces such as wastelands, artificially created habitats such as green roofs, and places where city-dwellers live and work such as grasslands within residential areas (MacIvor & Lundholm, 2011; Gunnarsson & Federsel, 2014). Although this kind of urban green infrastructure was probably not created with the intention to foster biodiversity in the first place, it can quantitatively make an important contribution to urban greenspace in general (Tewksbury et al., 2002), and thus, to the generation of habitat for pollinator species in particular.

A further drawback is that most studies analyzed responses of alpha-diversity measures (i.e., species richness) on urban matrix variables only (e.g., amount of impervious cover, Fortel et al., 2014; Threlfall et al., 2015; landscape composition, Baldock et al., 2015), while responses at the individual species level were rarely investigated. Although knowledge of shifts in species composition, and especially the occurrence of certain target species, is mandatory for reasonable conservation action plans, only a few studies hitherto assessed urbanization effects on species assemblages and single species (but see Bates et al., 2011; Banaszak-Cibicka & Żmihorski, 2012 for bee species; Hülsmann et al., 2015 for bumblebees).

Having these shortcomings in mind, our overriding aim was to analyze how wild bee species respond to various urban environmental parameters at two spatial scales: (i) the local habitat scale (i.e., site features related to management, design, plant species richness, flower density, restoration efforts); and (ii) the surrounding urban matrix (i.e., area size, isolation from similar habitats, urbanization level). In this context we used the urbanization score proposed by Seress et al. (2014) for the first time. We aimed on covering the response of (a) the alpha diversity of the bees (in terms of species richness and species occurences on the investigated sites), (b) the beta diversity of the bees (in terms of species turnover along the environmental gradients), and (c) the individual bee species on the environmental parameters. Unlike former studies, we deliberately included different qualitative and quantitative characteristics of the vegetation in our analyses that described the sampling sites of our pilot study in much detail.

We conducted our study in the district of Marzahn-Hellersdorf in Berlin, Germany, as it is characterized by a variety of urban green infrastructure, including a range of formal to informal greenspaces. Wastelands and extensive meadows that arose from wastelands by low management regime are interspersed within both large-scale residential and detached housing. Some of these sites were subject to restoration efforts aiming to increase native grassland species (Fischer et al., 2013; Fischer, von der Lippe & Kowarik, 2013). In particular, we aimed to: (1) investigate the extent to which urban grasslands within residential areas are used by bee assemblages as foraging habitats; (2) analyze how bee assemblages and individual bee species are affected by habitat and urban matrix variables; and finally (3) determine if grassland restoration on wasteland sites contributes to bee conservation in urban environments.

## Material and Methods

### Study area and study sites

**Figure 1:**
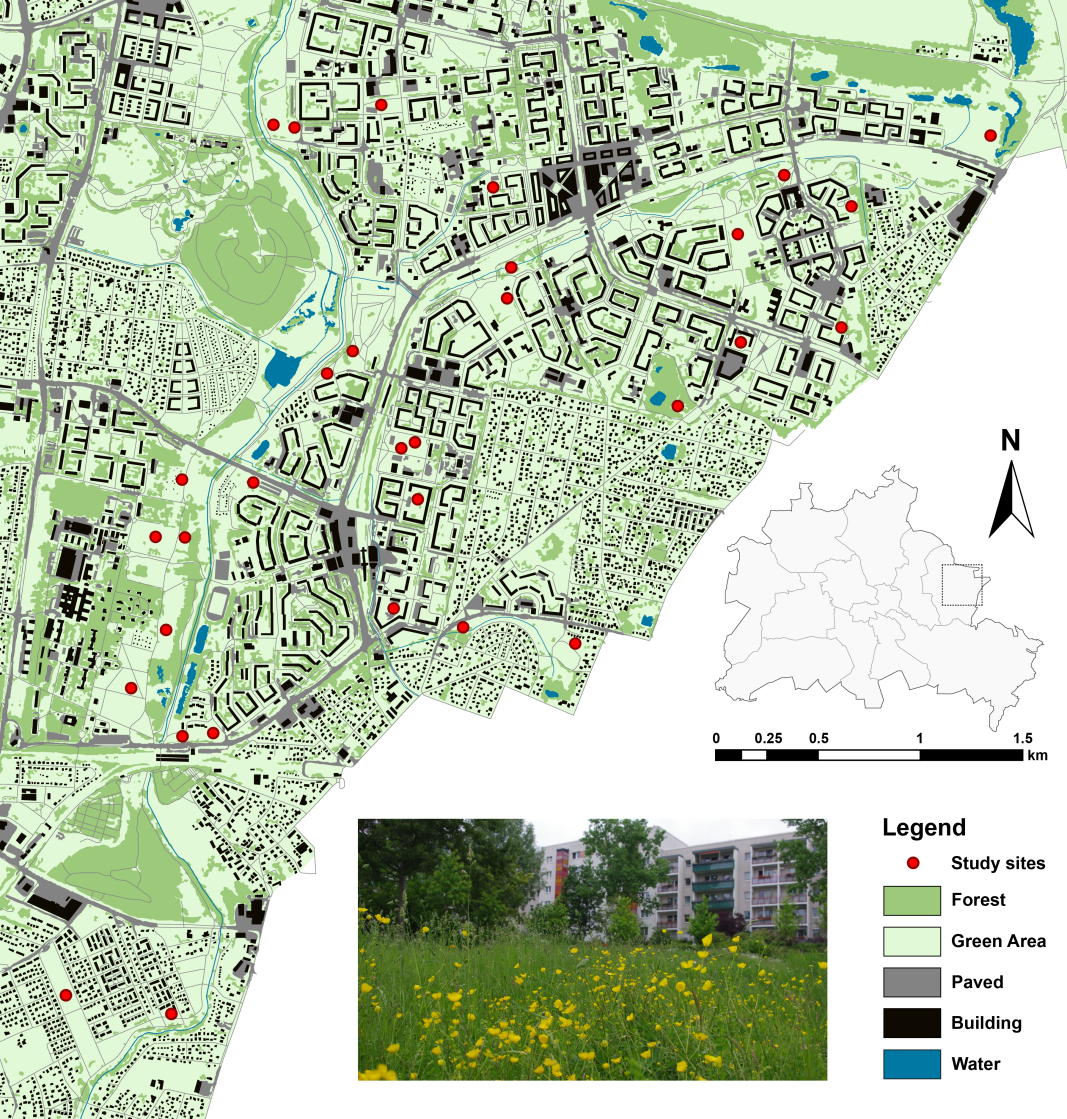
Study sites and landscape characteristics in Berlin/Marzahn-Hellersdorf (map was created on the basis of the Urban and Environmental Information System (UEIS), Berlin Department for Urban Development and the Environment).

All study sites were located in Marzahn-Hellersdorf (61.8 km^2^, 256,173 inhabitants), a district in the northeast of the German capital Berlin (Fig. 1). Large-scale residential housing areas built during the 1980s, but also featuring areas with detached housing and commercial areas, characterize the district’s landscape matrix. The provision with greenspaces in the study area is high as the public greenspaces in the district (1,262 ha, nearly 5% of the district’s surface), are accompanied by abundant privately owned greenspaces inside and outside the apartment blocks that are generally accessible to the public. These formal green spaces often consist of a mix of wooded areas and grasslands, including lawns and meadows, which are subject to high and low frequency of mowing, respectively. Moreover, extensive wastelands emerged due to infrastructure removal at the beginning of this millennium. These wastelands usually undergo only irregular or low management, and often, harbour ruderal grasslands and related types dominated by forbs. In order to reduce costs, most housing companies and public land owners perform a generally low management regime of these grasslands.

Within this context, we chose 30 study sites, well-defined by private and public greenspace property lines, and both inside and outside the apartment blocks. These sites were rather evenly distributed throughout the district and represented a range of open habitats: (1) wastelands with a mix of grassland and herbaceous vegetation; (2) ruderal grasslands; (3) infrequently mowed lawns; or (4) well-kept meadows and lawns. Additionally, some of the wasteland sites were previously subject to a restoration treatment to enhance the native grassland flora (for details, see Fischer et al., 2013; Fischer, von der Lippe & Kowarik, 2013). In the following, we address these types of greenspaces, dominated by grasses and forbs and managed at least irregularly, as grasslands.

### Bee sampling

Bees were sampled during two periods, one during early summer (15th and 16th of June) and one in midsummer (30th and 31st of July) in 2012 on sunny days without precipitation and maximum temperatures of at least 20 °C. Pan traps were used to collect the individuals. This is a common and efficient bee sampling method, well suited for all geographical regions and types of habitats (Leong & Thorp, 1999). Pan traps consisted of 15 cm Ø plastic bowls, which were spray painted in UV-bright yellow, white, and blue (Sparvar Leuchtfarbe; Spray-Color GmbH, Merzenich, Germany) and were filled with ∼300 ml of 4% formaldehyde solution as well as a drop of detergent to reduce surface tension. Three pan traps (one of each colour) were randomly positioned at each study site with at least 5 m distance from each other to avoid interference. In order to minimize strata collection bias, pan traps were attached to wood sticks 30 cm above the ground (Cane, Minckley & Kervin, 2001).

After two days, caught bees and bycatch were transferred into vials. Bee specimens were dried and identified to species level with the aid of a binocular microscope and standard identification keys. The European honeybee *Apis mellifera* was excluded from the list for all analysis since its abundance follows different seasonal patterns than wild bees (Tommasi et al., 2004) and its management in hives complicates the assessment (Cane, 2005).

### Environmental variables

**Figure 2:**
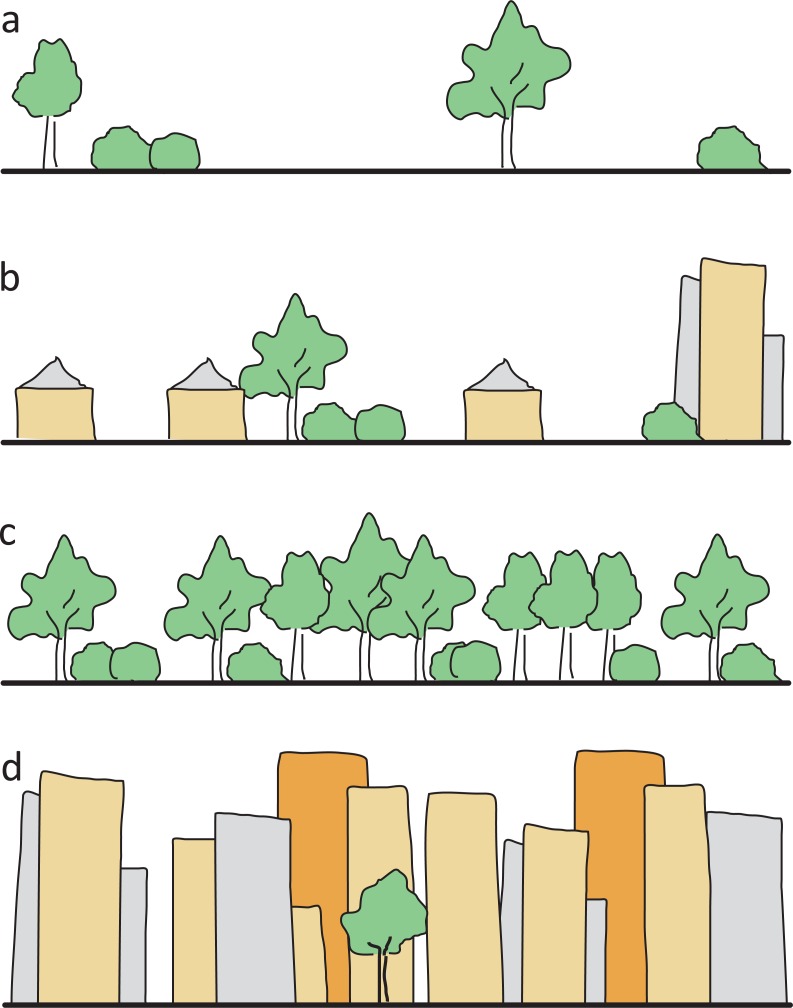
Illustration of the four isolation categories. (A) open site, grassland or parking lots, with only single shrubs/trees, no forest or high buildings as flight barriers within 100 m (0 isolation points), (B) single houses or allotments with built structures or few shrubs/trees as low flight barriers within 100 m (one isolation point), (C) dense forest and scrub (two isolation points), (D) row of high buildings as flight barriers (three isolation points).

To characterize study sites, we ascertained eight environmental variables (Table 1): (1) *Area size* in km^2^ for each site was measured using the free online tool Google Planimeter (http://www.acme.com/planimeter). (2) To reflect the degree of *urbanization* we used the ‘UrbanizationScore’ software developed by Seress et al. (2014). This program takes a 1 km^2^ area around each site and generates semi-automated scores based on the method by Czúni, Lipovits & Seress (2012). (3) Since past studies indicate that at least some bee species are affected not only by artificial barriers like buildings but also by natural barriers like forests (Bhattacharya, Primack & Gerwein, 2003; Farwig et al., 2009), the variable *isolation* was included. We calculated this variable based on the surrounding area of each cardinal direction (north, east, south, west) of the site’s borders. Each of the borders was sorted into one of four categories (Fig. 2) and was given respective points ranging from 0 to 3. The points of all four directions were added up to get a single isolation score for each site with 0 being the lowest, and 12 being the highest possible score. (4) The variable *management* reflected the mowing regime of a site and was differentiated between extensive (at least annually) and irregular mowing (less than once a year). Data on this variable was gained from respective authorities and additionally evaluated during field work. (5) As represented by the variable *restoration*, a third of all investigated sites had previously been part of a 5 year in-situ experiment to restore urban grassland vegetation on wasteland sites and thereby enhance the number of native plant species. The exact details of this experiment are given in Fischer et al. (2013) and Fischer, von der Lippe & Kowarik (2013). (6) The variable *site type* described the human influence on the layout of a site, that is, if and how a site was designed. This classification was based both on site history and the current status. We used the three categories ‘wastelands’ with no known intentional or directed design influence, and where vegetation was able to develop freely (e.g., after the complete destruction of infrastructure, and where the rubble was levelled out); ‘residual’ sites with only low design influence (e.g., vacant lots between houses where traces of past landscape design and plantings were still visible), and intentionally created, ‘designed’ sites (e.g., sites that included flower beds and lawns). (7) Mean degree of coverage with currently *flowering plants* was recorded during both bee-sampling periods to reflect the short term situation. Each site was put into one of four categories ranging from no visible flowers to maximum observed flowers. (8) The *number of plant species* was derived from records of the restoration experiment mentioned above (Fischer et al., 2013; Fischer, von der Lippe & Kowarik, 2013) and associated research activity in the other plots.

**Table 1:** Environmental variables of urban grassland sites used as predictor variables for the statistical analyses.

Variable	Mean (min/max)	Scale/Categories	Explanation
**Urban matrix**			
Area size	0.01 (0.001/0.07)	km^2^	Area as defined by the lot’s property lines, its bordering by housing or other infrastructure
Urbanization	0.000001 (−3,395/3,182)	Urbanization score	As of Seress et al. (2014)
Isolation	–	0–12	Continual score from 0 (open site) to 12 (isolated site); see Fig. 2
**Site variables**			
Management	–	Extensive mowing	Reflects the mowing regime of a site; differentiation was made between extensive (annually) and irregular mowing (less than once a year); data obtained from respective authorities and evaluated during field work
		Irregular mowing	
Restoration	–	No	To describe whether the site had previously been part of a five year in-situ experiment to restore grassland vegetation on wasteland sites to enhance the number of typical, native plant species. For details see Fischer et al. (2013) and Fischer, von der Lippe & Kowarik (2013)
		Yes	
Site type	–	*Wasteland site*, with no known intentional or directed human design influence, and where successional vegetation was able to develop freely	To describe the human influence on the layout/design of a site
		*Residual site* with low or no design influence (e.g., vacant lots between houses where traces of past landscape design and greening were visible)	
		*Designed site*, site that was purposefully constructed e.g., with formal elements like lawns, flower beds	
**Vegetation variables**			
Flower coverage	–	0–25% (1)	Mean degree of coverage with currently flowering plants (excluding grasses); recorded during both bee sampling periods to reflect the short term situation; categories range from (1) no visible flowers to (4) maximum observed flowers
		25–50% (2)	
		50–75% (3)	
		75–100% (4)	
Plant species	27 (6/69)	Number of plant species	As of Fischer et al. (2013) and Fischer, von der Lippe & Kowarik (2013) and associated research activity

### Statistical analysis

One sample unit includes the pooled data from all three pan traps per site and both sampling periods. To detect groupings of sample sites, the bee community’s response to environmental variables (Table 1) was tested using non-metric multidimensional scaling (NMDS) as multivariate analysis (McCune & Grace, 2002). In order to enhance accuracy of statistical analyses and to reduce the statistical noise, bee species detected only once or twice were omitted from the data set beforehand (McCune & Grace, 2002). We also excluded *Apis mellifera*, which occurred at all study sites. Thus, 37 bee species, with 1,246 individuals totally, were subjected to NMDS. Relative abundances of these species were standardized performing Wisconsin double standardization and square root transformation. Bray–Curtis dissimilarity matrix of bees was used as base for the ordination. A maximum number of 100 random starts were conducted in search for a stable solution. Environmental variables were fitted onto the ordination with 10,000 permutations to assess significance of correlations between species and environmental data.

To find the distinctive species between restored and unrestored sites as well as among the three categories of urbanization (low = −3.4–−1.27, medium = −1.26–1.67, high = 1.68–3.18), a similarity percentage (SIMPER) analysis was conducted. The SIMPER analysis performs pairwise comparisons of groups of sampling units and finds the average contribution of each species to the average overall Bray–Curtis dissimilarity (Clarke, 1993). The function displays the most important species, which contribute to at least 70% of the differences between groups. The level of significance was *P* < 0.05.

Finally, individual responses of the distinctive bee species (nine species from SIMPER analyses) to all environmental variables were analyzed using Poisson generalized linear models (GLM). Due to overdispersion, standard errors were corrected using a quasi-Poisson distribution. The most appropriate models were selected by means of analysis of deviance. As a goodness-of-fit measure the pseudo R^2^ based on null and residual deviance was calculated (Zuur et al., 2009). All statistical analyses were performed using the R software environment (version 3.0.1, R Core Team, 2013) including package VEGAN (version 2.0-9, Oksanen et al., 2013).

## Results

In all, 62 bee species were collected with 1,706 specimens (Appendix S1). Of these, 38 species were sampled with three or more individuals. The most abundant species was the European honey bee (*Apis mellifera*) with 430 specimens, which therefore accounted for about 25% of all counts. Other common species were *Lasioglossum pauxillum* (359 individuals), *L. morio* (223), *Dasypoda hirtipes* (141) and *Andrena flavipes* (66).

### Structure of bee assemblage

**Figure 3:**
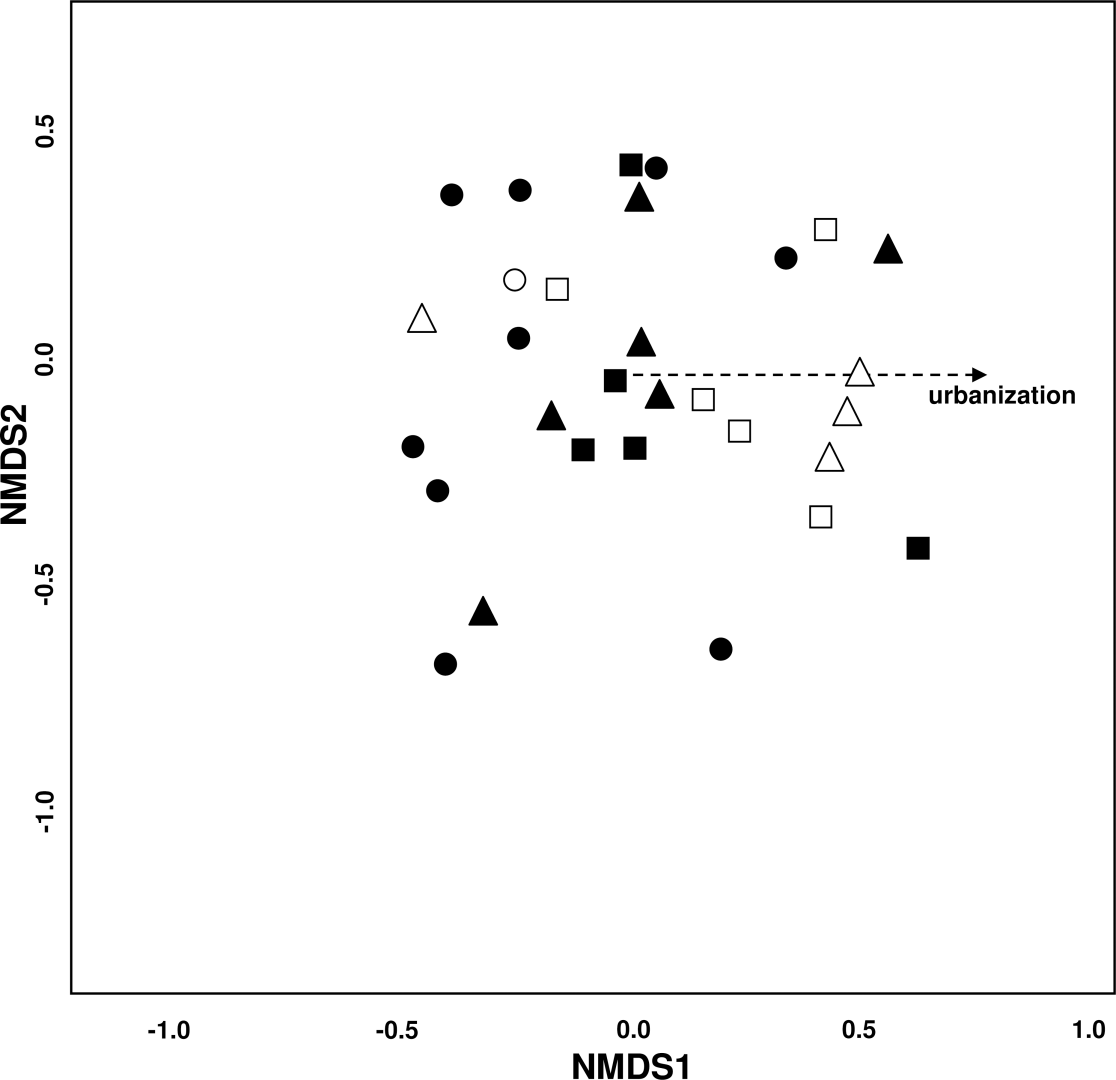
Variation of bee species assemblages among different levels of urbanization and restoration. Non-metric multidimensional scaling (NMDS) ordination (stress = 0.19). Environmental variables fitted onto the ordination plot: dotted line: *P* = 0.05−0.1. Symbols for study sites: site with ● = low (−3.4 –−1.27), ▴ = medium (−1.26–1.67), and ■ = high (1.68–3.18) urbanization degree. Empty symbols incidicate sites that have undergone restoration measures.

The NMDS plot (stress = 0.19) showed no clear groupings of the study sites (Fig. 3). Urbanization significantly affected species composition (*P* = 0.037) while all other environmental variables had no effect. Matching this underlying environmental gradient, sites with a higher urbanization value were located more to the right of the plot and likewise sites with a low urbanization value were located more to the left—indicating differences in species distribution among the three urbanization levels.

SIMPER analysis revealed that five species (*Bombus lapidarius*, *B. terrestris*, *Colletes similis*, *Dasypoda hirtipes* and *Halictus subauratus*) seem to thrive well in highly urbanized sites while four species (*Andrena flavipes*, *Bombus pascuorum, Lasioglossum malachurum* and *L. villosulum*) appeared to be sensitive to urbanization (Table 2).

### Species responses to environmental variables

**Figure 4:**
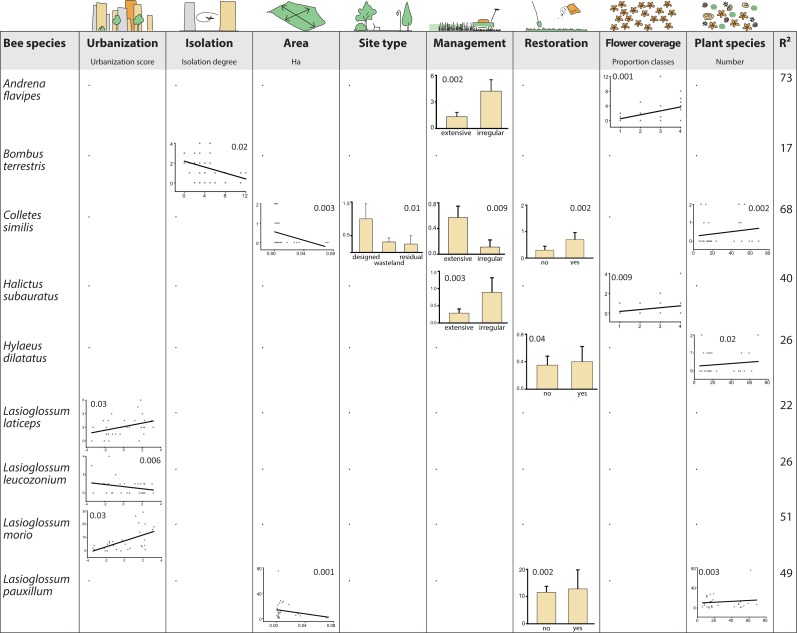
Bee species as winners or loosers in residential areas of Berlin. Shown are responses of bee species, which are significantly affilated to different levels of urbanization (SIMPER analysis, Table 2) to environmental variables using general linear models (GLM) for analysis. Only species with significant responses are displayed (inserted numbers show *P* values of responses). Species without significant results: *Bombus lapidarius*, *Bombus pascuorum*, *Dasypoda hirtipes*, *Halictus rubicundus*, *Halictus tumulorum*, *Hylaeus hyalinatus*, *Lasioglossum calceatum*,* Lasioglossum malachurum,* and *Lasioglossum villosulum*.

Results from the GLM indicated that species showed multidirectional responses to environmental variables (Fig. 4). A consistent trend was not recognizable, and environmental variables did not exceed three species responses each. Some of the species showed even contrasting responses but restoration and flower density had overall positive effects. In contrast, area and isolation had a negative impact on tested species. All other variables were related to increasing or decreasing abundances, depending on the species, and even varying within the same genera. However, urbanization, management and plant species richness had more positive than negative effects.

**Table 2:** Affiliation of bee species to different levels of urbanization. Results of SIMPER analyses (*P* < 0.05). ‘x’ indicates in which of the compared categories (urbanization: low vs. medium vs. high urbanization scores) the respective species had the higher average abundance.

Bee species	Urbanization		
	Low	Medium	High
*Andrena flavipes*	x	⋅	⋅
*Bombus lapidarius*	⋅	⋅	x
*Bombus pascuorum*	x	⋅	⋅
*Bombus terrestris*	⋅	⋅	x
*Colletes similis*	⋅	x	x
*Dasypoda hirtipes*	⋅	⋅	x
*Halictus rubicundus*	⋅	x	⋅
*Halictus subauratus*	⋅	x	x
*Halictus tumulorum*	⋅	x	⋅
*Hylaeus dilatatus*	x	⋅	x
*Hylaeus hyalinatus*	⋅	x	⋅
*Lasioglossum calceatum*	⋅	x	⋅
*Lasioglossum laticeps*	⋅	x	⋅
*Lasioglossum leucozonium*	⋅	x	⋅
*Lasioglossum malachurum*	x	⋅	⋅
*Lasioglossum morio*	⋅	x	⋅
*Lasioglossum pauxillum*	⋅	x	⋅
*Lasioglossum villosulum*	x	⋅	⋅

## Discussion

With 62 bee species, more than 20% of taxa known for Berlin (Saure, 2005) were found during the study. This result is remarkable, considering that only one sampling method was used, the sampling period was relatively short and only one district of Berlin has been taken as model area for this pilot study. In this context, it is noteworthy that due to late sampling time we may have underestimated species that are more abundant in early season and so species inventory could be extended in further studies. The study’s species inventory was comparable to those of more rural and natural grasslands of Berlin (Dathe, 1969; Saure, 1993). The investigated urban grasslands were not only suitable for common bee species, but also for rare taxa like *Anthophora aestivalis* and *Lasioglossum quadrinotatum* or endangered species such as *Halictus subauratus* and *Lasioglossum malachurum* (Eichfeld & Buchholz, 2014). The fact that only few species were really abundant (e.g., *Dasypoda hirtipes*, *Lasioglossum morio*, *L. pauxillum*) while the majority of taxa occurred only with lower individual sums is typical for bee assemblages, independent from an urban or rural context (Cane, 2005).

Thus, our results clearly confirmed findings that cities provide foraging habitats for wild bees (Paker et al., 2014; Baldock et al., 2015; Threlfall et al., 2015). With our study, we extend this knowledge by demonstrating for the first time that not only flower-rich gardens or park meadows are valuable urban habitats for insects (e.g., Ahrné, Bengtsson & Elmqvist, 2009; Garbuzov, Ratnieks & Thompson, 2013) but also differently structured and managed grasslands in residential areas.

### (a) Minor effect of urban matrix variables

Urbanization significantly affected species composition. This can be explained by a species turnover as the SIMPER analysis (Table 2) showed that some species seemed to thrive very well in urban sites (*Bombus lapidarius*, *B. terrestris*, *Colletes similis*, *Dasypoda hirtipes* and *Halictus subauratus*) while others negatively responded to urbanization (*Andrena flavipes*, *Bombus pascuorum*, *Lasioglossum malachurum* and *L. villosulum*). Thus, urbanization did not seem to have an overall negative impact, but it affected urbanization winners and losers in different ways as suggested by previous studies (e.g., Banaszak-Cibicka & Żmihorski, 2012; Hülsmann et al., 2015). Correspondingly, Turrini & Knop (2015) found that urbanization also favors specific species. Community composition may be determined by stochasticity (Sattler et al., 2010), and urbanization impacts depend on geographical, historical and economic factors of cities (McKinney, 2008). Our results and those of recent studies indicate that urbanization can also have positive effects for certain species.

In our study, most of the detected bee species were not affected by any of the environmental variables while few showed multidirectional responses (e.g., *Colletes similis*, *Lasioglossum pauxillum*). Matrix variables were found to be important for structuring pollinator communities and explaining patterns of species richness, abundance and biotic interactions in rural landscapes (Steckel et al., 2014; Clough et al., 2014). Matrix variables (urbanization, isolation, area) affected only a few species, and in different ways:

- Urbanization had a negative effect only for one species, *Lasioglossum leucozonium*, although this species is known to occur in a broad spectrum of rural as well as urban habitat types (Saure, 2005).
- Similarly, isolation negatively affected only *Bombus terrestris,* which is surprising because this species is known to be one of the most abundant bumblebee species in many rural and urban habitats of Europe (Parmentier et al., 2014; Hülsmann et al., 2015). However, since the goodness-of-fit measure for this model was quite low (R^2^ = 17, Fig. 4), results should not be overestimated. In fact, it appears that isolation plays a minor role for bees that might easily move between their foraging habitats due to their high mobility (Zurbuchen et al., 2010; Krewenka et al., 2011).
- Only two species were affected by area size, being more abundant in smaller areas. Size of green area can have positive effects on bee assemblages (Henning & Ghazoul, 2011) but other studies did not find size effects in cities at least for bumblebees (Gunnarsson & Federsel, 2014). In our study, responses of only two species, *Colletes similis* and *Lasioglossum pauxillum*, may therefore be explained by stochasticity or undetected side effects.

### (b) Positive effect of restoration

Site variables (site type, management, restoration) affected several species, and restoration had an overall positive effect on species abundances. This is substantiated by the results from the SIMPER analysis, which revealed that 11 species were strongly associated to sites with restoration (Appendix S1). From these results we conclude that restoration can significantly shape bee assemblages in urban environments (see below).

### (c) Positive effect of vegetation variables

Flower density and number of plant species had an overall positive effect on bee species as shown in other studies in churchyards and cemeteries (Bates et al., 2011), parks (Matteson, Grace & Minor, 2013) and urban habitats in general (Henning & Ghazoul, 2012; Hülsmann et al., 2015).

Restoration was previously determined to have positive effects on bee species in rural areas, e.g., due to increased plant diversity (Albrecht et al., 2007). Our study demonstrates that restoration of grassland on urban wasteland sites increased the abundance of wild bees in urban settings as well. Regarding the vegetation of the sites, the restoration treatments increased plant species richness by adding a considerable pool of native dry-grassland species (Fischer, von der Lippe & Kowarik, 2013) and resulted in an increased floral diversity (Senße, 2013). This is in line with a study that revealed the positive effect of increased floral diversity for bumblebee and hoverfly abundances in amenity grassland (Blackmore & Goulson, 2014) and findings of Wastian, Unterweger & Betz (2016). Whereas Hanley, Awbi & Franco (2014) showed for urban gardens that bumblebees have no dietary preference for native or non-native plants, there is also evidence from highly disturbed sites (roadverges), where restoration of the native vegetation especially resulted in increased bee richness (Hopwood, 2008).

## Practical Implications

We are aware that our conclusions are drawn from a snapshot since data were collected only in a single group of habitats (i.e., different types of grassland in residential areas) during a rather short time. However, keeping in mind that further studies will deepen these insights, our pilot study indicates that urban grasslands in general provide new foraging habitats for pollinators, and that in particular sites that had undergone restoration treatments were favoured by some wild bee species. As management itself was not found to be an important determinant for the bee species assemblages, we assume that the mix of differently managed sites in a neighbourhood may promote the occurrence of the species assemblages found. This demonstrates that opposed to the global trend of urban landscapes with uniform, highly managed lawns (Ignatieva et al., 2015), the variety in (grassland) habitats matters for species conservation in cities. We therefore encourage to include urban areas in initiatives of pollinator conservation programmes, and especially to include also informal greenspaces. These may foster an additional set of species compared to formal greenspaces such as gardens and parks. By applying effective restoration measures on underused wasteland sites, valuable substitute habitats can be created—a win–win situation for the urban fauna *and* flora that can still benefit from low costs by extensive management regimes.

##  Supplemental Information

10.7717/peerj.2729/supp-1 Appendix S1 Species list List of bee species caught in 30 study sites in Marzahn-Hellersdorf/Berlin during early summer (15th and 16th of June) and midsummer (30th and 31st of July) in 2012 (nomenclature followed Westrich et al., 2008). Explanations: **R+** = Bee species significantly affiliated to restoration (SIMPER analysis, *P* < 0.05).

10.7717/peerj.2729/supp-2 Data S1 Raw Data for Statistics

## Additional Information and Declarations

### Data Availability

The following information was supplied regarding data availability: The raw data has been supplied as a Supplemental File.
