# Supplementary material for: Disentangling urban habitat and matrix effects on wild bee species
Source: PeerJ. 2016 Nov 29;4:e2729. doi: 10.7717/peerj.2729 (PMC5131621; doi:10.7717/peerj.2729)
Supplement: Appendix S1 — List of bee species caught in 30 study sites in Marzahn-Hellersdorf/Berlin during early summer (15th and 16th of June) and midsummer (30th and 31st of July) in 2012 (nomenclature followed Westrich et al., 2008). Explanations: R+ = Bee species significantly affiliated to restoration (SIMPER analysis, P < 0.05). [file peerj-04-2729-s001.doc]

**Appendix S1:** List of bee species caught in 30 study sites in Marzahn-Hellersdorf/Berlin during early summer (15th and 16th of June) and midsummer (30th and 31st of July) in 2012 (nomenclature followed Westrich *et al*., 2008). Explanations: **^R+^** = Bee species significantly affiliated to restoration (SIMPER analysis, P < 0.05).

| **Species** | **1** | **2** | **3** | **4** | **5** | **6** | **7** | **8** | **9** | **10** | **11** | **12** | **13** | **14** | **15** |
| --- | --- | --- | --- | --- | --- | --- | --- | --- | --- | --- | --- | --- | --- | --- | --- |
| *Andrena bicolor* | . | . | . | . | . | . | . | . | . | . | . | . | . | . | . |
| *Andrena dorsata* | . | . | . | . | 1 | . | 1 | . | 1 | . | . | . | . | . | . |
| *Andrena flavipes* **^R+^** | 1 | 12 | . | 4 | 8 | . | 5 | 2 | 3 | . | 1 | 4 | 1 | 6 | 2 |
| *Andrena minutula* | . | . | . | . | . | . | . | 3 | . | . | . | 1 | . | . | . |
| *Andrena minutuloides* | . | . | . | . | . | . | . | . | 1 | . | . | . | . | . | . |
| *Andrena nigroaenea* | . | 1 | . | . | . | . | . | . | . | . | . | . | . | . | 1 |
| *Andrena nigrospina* | . | . | . | . | . | . | . | . | . | . | . | . | . | . | . |
| *Andrena nitida* | . | . | . | . | . | . | . | . | . | . | . | . | . | . | . |
| *Anthidium manicatum* | . | . | . | . | . | . | . | . | . | . | . | . | . | . | . |
| *Anthidium oblongatum* | . | . | . | . | . | . | . | . | . | . | . | . | . | . | . |
| *Anthidium punctatum* | . | . | 1 | . | . | . | . | . | . | . | . | . | . | . | . |
| *Anthophora aestivalis* | . | . | . | . | 1 | . | . | . | . | . | . | . | . | . | . |
| *Apis mellifera* | 12 | 11 | 18 | 5 | 10 | 9 | 6 | 23 | 36 | 30 | 18 | 8 | 5 | 15 | 19 |
| *Bombus hortorum* | . | . | . | . | . | . | . | . | . | . | . | . | . | . | . |
| *Bombus hypnorum* | . | . | . | . | . | . | . | . | . | . | . | . | . | . | 2 |
| *Bombus lapidarius* | . | 1 | . | . | 1 | 1 | . | 2 | 1 | . | 1 | . | . | . | 2 |
| *Bombus lucorum* | . | . | . | . | . | . | . | 1 | . | 1 | . | . | . | . | . |
| *Bombus pascuorum* **^R+^** | 1 | 2 | . | . | 1 | . | 2 | . | . | 3 | . | 2 | . | 2 | . |
| *Bombus rupestris* | . | 1 | . | 2 | . | . | . | . | . | . | . | . | . | 1 | . |
| *Bombus sylvarum* | . | . | . | . | . | . | . | . | 1 | 1 | . | . | . | . | 2 |
| *Bombus terrestris* **^R+^** | 2 | 2 | 1 | 1 | . | 1 | 3 | 3 | 3 | . | 1 | 3 | 1 | 1 | . |
| *Bombus vestalis* | . | . | . | . | . | . | . | . | 1 | . | . | . | . | . | . |
| *Colletes similis* | . | . | 1 | . | . | . | . | . | . | . | . | . | 1 | . | . |
| *Dasypoda hirtipes* | . | 2 | 2 | 2 | 6 | 2 | 6 | 1 | 1 | 2 | 10 | 10 | 5 | 2 | 3 |
| *Halictus quadricinctus* | 1 | . | . | . | . | . | . | . | . | . | . | . | . | . | . |
| *Halictus rubicundus* | . | . | . | . | . | 1 | 1 | . | . | 2 | . | 1 | . | 1 | . |
| *Halictus sexcinctus* | . | . | . | . | . | . | . | . | . | . | 4 | . | . | . | . |
| *Halictus subauratus* **^R+^** | . | . | 1 | . | 1 | . | 1 | . | 1 | . | . | . | . | . | 1 |
| *Halictus tumulorum* | 1 | . | 1 | . | . | . | 1 | 2 | . | 1 | . | . | 2 | 1 | 1 |
| *Hylaeus brevicornis* | . | . | . | . | . | . | . | . | . | . | . | . | . | . | . |
| *Hylaeus cardioscapus* | . | . | . | . | . | . | . | . | . | 1 | . | . | . | . | . |
| *Hylaeus communis* | . | . | . | . | . | . | . | . | . | . | . | . | 1 | 1 | . |
| *Hylaeus confusus* | . | . | . | . | . | . | . | . | . | 1 | . | . | . | . | . |
| *Hylaeus annularis* | 1 | . | . | . | 2 | . | . | . | . | 1 | 1 | 1 | . | . | . |
| *Hylaeus hyalinatus* **^R+^** | . | . | 2 | . | . | . | . | . | . | . | . | . | . | 6 | . |
| *Lasioglossum calceatum* **^R+^** | 2 | 1 | 5 | 1 | 3 | 1 | 3 | 1 | 2 | . | 1 | 2 | 1 | 2 | 4 |
| *Lasioglossum laticeps* | 1 | 1 | 2 | 2 | 2 | 2 | 5 | 3 | 1 | 2 | 1 | . | 2 | 3 | 3 |
| *Lasioglossum leucozonium* **^R+^** | . | . | . | . | . | 1 | 1 | 1 | 1 | . | 2 | 4 | 1 | 1 | . |
| *Lasioglossum malachurum* **^R+^** | . | 3 | 11 | . | . | . | 1 | . | . | . | . | . | . | . | . |
| *Lasioglossum morio* **^R+^** | 4 | 4 | 1 | 3 | 18 | 8 | 21 | 2 | . | 1 | 1 | 4 | 20 | 16 | 1 |
| *Lasioglossum pauxillum* **^R+^** | 1 | 2 | 7 | 2 | 7 | 5 | 22 | 25 | 4 | 10 | 2 | 9 | 12 | 75 | 21 |
| *Lasioglossum quadrinotatum* | . | . | . | . | . | . | . | . | . | . | . | . | 1 | . | . |
| *Lasioglossum sexnotatum* | . | . | . | . | . | . | . | . | . | . | . | . | . | . | . |
| *Lasioglossum sexstrigatum* | . | . | 1 | . | . | . | 1 | 1 | . | . | . | . | . | 2 | . |
| *Lasioglossum villosulum* **^R+^** | . | 1 | 3 | . | 1 | . | . | . | . | . | . | 1 | . | 2 | . |
| *Macropis europaea* | . | . | . | . | . | . | . | . | . | . | . | . | . | . | . |
| *Megachile circumcincta* | 1 | 1 | . | . | 1 | . | . | . | . | . | . | . | . | . | . |
| *Megachile ericetorum* | 1 | . | 3 | 1 | . | . | . | . | . | . | . | . | . | . | . |
| *Megachile maritima* | . | . | . | . | . | . | . | . | . | . | . | . | . | 2 | . |
| *Megachile rotundata* | . | . | . | . | . | . | 1 | . | . | . | . | . | . | . | . |
| *Megachile willughbiella* | . | . | 1 | . | . | . | . | . | . | . | . | . | . | . | . |
| *Melitta haemorrhoidalis* | . | . | . | . | . | . | . | . | . | . | 1 | . | . | . | . |
| *Osmia adunca* | . | . | . | . | . | . | . | . | . | . | . | . | . | . | . |
| *Osmia aurulenta* | . | . | 1 | . | . | . | . | . | . | . | . | . | . | . | . |
| *Osmia crenulata* | . | . | . | . | . | . | . | . | . | . | . | . | . | . | . |
| *Osmia leucomelana* | . | . | . | . | . | . | . | . | . | 2 | . | . | . | . | . |
| *Osmia mustelina* | . | . | . | . | . | . | . | . | . | . | . | . | . | . | . |
| *Osmia rapunculi* | . | . | . | . | . | . | . | . | . | . | . | . | . | . | . |
| *Osmia spinulosa* | . | 4 | 1 | . | . | . | 3 | 1 | . | . | . | . | . | . | . |
| *Panurgus calcaratus* | . | . | . | . | . | 2 | . | . | . | . | . | . | . | . | . |
| *Sphecodes ephippius* | . | . | . | 1 | . | . | . | . | . | . | . | . | . | . | . |
| *Systropha curvicornis* | . | . | . | . | . | . | . | . | . | . | . | . | . | . | . |

| **Species** | **16** | **17** | **18** | **19** | **20** | **21** | **22** | **23** | **24** | **25** | **26** | **27** | **28** | **29** | **30** |
| --- | --- | --- | --- | --- | --- | --- | --- | --- | --- | --- | --- | --- | --- | --- | --- |
| *Andrena bicolor* | . | . | . | 1 | . | . | 1 | . | . | . | . | . | . | . | . |
| *Andrena dorsata* | . | . | . | . | . | . | . | . | . | . | . | . | . | . | . |
| *Andrena flavipes* **^R+^** | . | 8 | . | 1 | . | 5 | . | . | . | . | . | . | . | . | 3 |
| *Andrena minutula* | . | . | 1 | . | . | . | 1 | . | . | . | . | . | . | . | . |
| *Andrena minutuloides* | . | . | . | . | . | . | . | . | . | . | . | . | . | . | . |
| *Andrena nigroaenea* | . | . | . | . | . | . | . | . | . | . | . | . | . | . | . |
| *Andrena nigrospina* | . | . | . | . | . | . | 1 | . | . | . | . | . | . | . | 2 |
| *Andrena nitida* | . | . | . | . | . | . | . | . | . | . | . | . | . | . | 1 |
| *Anthidium manicatum* | 1 | . | . | . | . | . | . | . | . | . | . | . | . | . | . |
| *Anthidium oblongatum* | . | . | . | . | . | . | . | . | . | . | . | . | . | 1 | . |
| *Anthidium punctatum* | . | . | . | . | . | . | . | . | . | . | . | . | . | . | . |
| *Anthophora aestivalis* | . | . | . | . | . | . | . | . | . | . | . | . | . | . | . |
| *Apis mellifera* | 17 | 6 | 9 | 27 | 13 | 15 | 7 | 9 | 23 | 10 | 9 | 6 | 7 | 27 | 20 |
| *Bombus hortorum* | . | . | . | . | . | . | 1 | 1 | . | 1 | 1 | 1 | . | . | . |
| *Bombus hypnorum* | . | . | . | . | . | . | . | . | . | . | . | . | . | . | 1 |
| *Bombus lapidarius* | . | . | . | 2 | 1 | 2 | 3 | 3 | 2 | 2 | . | . | . | 1 | . |
| *Bombus lucorum* | . | . | . | . | 1 | . | . | . | 1 | . | . | . | . | . | . |
| *Bombus pascuorum* **^R+^** | . | . | . | . | 1 | . | . | . | . | 1 | . | . | . | 1 | . |
| *Bombus rupestris* | . | 2 | . | 1 | . | . | . | . | . | . | 1 | . | . | . | . |
| *Bombus sylvarum* | . | . | . | . | . | . | . | 1 | . | 1 | . | . | . | . | . |
| *Bombus terrestris* **^R+^** | . | . | 1 | 2 | . | 4 | 2 | 1 | 2 | 1 | 3 | . | 4 | 2 | 2 |
| *Bombus vestalis* | . | . | . | . | . | . | . | . | . | . | . | . | . | . | . |
| *Colletes similis* | 1 | 2 | 2 | . | . | . | . | . | . | 2 | . | 1 | 1 | . | 2 |
| *Dasypoda hirtipes* | 5 | 3 | 1 | 3 | 6 | 12 | 3 | . | 1 | 5 | 11 | . | 3 | 31 | 3 |
| *Halictus quadricinctus* | . | . | . | . | . | 1 | 1 | . | 1 | . | . | . | . | . | 2 |
| *Halictus rubicundus* | 1 | 2 | 1 | 6 | 1 | 3 | . | 2 | . | . | 1 | . | 1 | 4 | . |
| *Halictus sexcinctus* | . | 1 | . | . | . | . | . | . | . | . | . | . | . | . | 1 |
| *Halictus subauratus* **^R+^** | 1 | 1 | 2 | . | . | 4 | . | . | . | . | 1 | . | . | . | . |
| *Halictus tumulorum* | . | . | 2 | 1 | . | 1 | . | . | . | . | . | 1 | . | 1 | . |
| *Hylaeus brevicornis* | . | . | . | . | . | . | . | . | . | . | . | . | . | 1 | . |
| *Hylaeus cardioscapus* | . | . | . | . | . | . | . | . | . | . | . | . | . | . | . |
| *Hylaeus communis* | . | . | . | . | . | . | . | . | . | . | . | . | . | . | . |
| *Hylaeus confusus* | . | . | . | . | . | . | . | 3 | . | . | . | . | . | . | . |
| *Hylaeus annularis* | . | . | . | 1 | . | . | . | 2 | . | . | . | 1 | . | 1 | . |
| *Hylaeus hyalinatus* **^R+^** | 1 | 1 | . | . | . | . | . | . | . | . | . | 1 | . | . | 1 |
| *Lasioglossum calceatum* **^R+^** | 3 | 6 | 1 | 6 | 2 | 1 | 3 | . | 1 | 5 | 1 | 1 | 3 | 2 | . |
| *Lasioglossum laticeps* | . | 3 | 4 | 6 | 3 | 3 | . | 4 | . | 1 | 2 | 2 | 2 | 1 | 2 |
| *Lasioglossum leucozonium* **^R+^** | . | 1 | . | 1 | 1 | 1 | 1 | . | 3 | 1 | . | . | . | . | . |
| *Lasioglossum malachurum* **^R+^** | . | . | . | . | . | . | . | . | . | . | 4 | . | . | . | . |
| *Lasioglossum morio* **^R+^** | 14 | 26 | 29 | 4 | 7 | 5 | . | 7 | 2 | 6 | 4 | 5 | 8 | . | 2 |
| *Lasioglossum pauxillum* **^R+^** | 24 | 9 | 4 | 28 | 2 | 27 | 13 | 8 | 16 | 5 | 3 | 13 | 1 | . | 2 |
| *Lasioglossum quadrinotatum* | . | . | . | . | . | . | . | . | . | . | . | . | . | . | . |
| *Lasioglossum sexnotatum* | . | . | . | . | . | . | . | . | . | . | . | . | . | . | . |
| *Lasioglossum sexstrigatum* | . | . | . | 1 | . | 1 | . | . | 1 | . | . | 1 | . | . | . |
| *Lasioglossum villosulum* **^R+^** | . | . | . | . | . | . | . | . | 1 | 2 | 1 | . | . | . | . |
| *Macropis europaea* | . | . | . | . | . | . | . | . | . | 1 | . | . | . | . | . |
| *Megachile circumcincta* | . | . | . | . | . | . | . | . | . | . | . | . | . | . | . |
| *Megachile ericetorum* | . | . | 1 | . | . | 1 | . | . | . | . | 1 | . | . | . | . |
| *Megachile maritima* | . | . | . | . | . | . | . | . | . | 1 | . | . | . | . | . |
| *Megachile rotundata* | . | . | . | . | . | . | . | . | . | . | . | . | . | 1 | . |
| *Megachile willughbiella* | . | . | . | . | 2 | . | . | . | . | . | . | . | . | . | . |
| *Melitta haemorrhoidalis* | 1 | . | . | 1 | 1 | . | . | . | . | . | . | . | . | . | . |
| *Osmia adunca* | . | . | . | . | . | . | . | . | . | . | . | . | 1 | . | . |
| *Osmia aurulenta* | . | . | . | . | . | . | . | . | . | . | . | . | . | . | . |
| *Osmia crenulata* | . | . | . | . | . | 1 | . | . | . | . | . | . | . | 1 | . |
| *Osmia leucomelana* | . | . | . | . | . | . | . | . | . | . | . | . | . | . | . |
| *Osmia mustelina* | . | . | . | . | 1 | . | . | . | . | . | . | . | . | . | . |
| *Osmia rapunculi* | . | . | . | . | . | . | . | . | . | . | 1 | . | . | . | . |
| *Osmia spinulosa* | . | . | . | . | . | . | . | . | . | 1 | . | . | . | . | . |
| *Panurgus calcaratus* | . | . | . | . | . | . | . | . | . | . | . | . | . | . | . |
| *Sphecodes ephippius* | . | . | . | . | . | . | . | . | . | . | . | . | . | . | . |
| *Systropha curvicornis* | . | . | . | . | . | . | . | . | . | . | . | . | . | 3 | . |
